# O Descenso Noturno Atenuado pode ser um Preditor de Gravidade e Complexidade da Doença Arterial Coronariana em Pacientes Internados com Síndrome Coronariana Aguda?

**DOI:** 10.36660/abc.20220401

**Published:** 2022-07-07

**Authors:** Andrea Pio-Abreu

**Affiliations:** 1 Universidade de São Paulo Unidade de Hipertensão Arterial da Disciplina de Nefrologia São Paulo SP Brasil Unidade de Hipertensão Arterial da Disciplina de Nefrologia, Universidade de São Paulo (USP), São Paulo, SP – Brasil

**Keywords:** Pressão Arterial, Descenso Noturno, Hipertensão Arterial, Síndrome Coronariana Aguda, Doença Arterial Coronariana

Está bem estabelecido que há implicações prognósticas significativas decorrentes do comportamento anormal de alguns parâmetros da pressão arterial (PA) nas 24 horas, obtidos através da Monitorização Ambulatorial de Pressão Arterial (MAPA).^1^ Dentre eles, destacam-se os comportamentos anormais das médias da pressão arterial sistólica (PAS) e diastólica (PAD) nas 24 horas, vigília e sono.^2^ Especificamente em relação ao comportamento da PA durante o sono, já está bem estabelecido que a ausência do descenso noturno possui, de forma independente, impacto significativo no aumento do risco cardiovascular.^3,4^ Outros parâmetros que podem ter implicações prognósticas, são a elevação matinal precoce da PA, e a maior variabilidade da PA nas 24 horas.^1,5^

Algumas recomendações são essenciais para o sucesso do exame da MAPA, dentre elas, a orientação para que o paciente mantenha suas atividades habituais durante o dia de monitorização. Neste sentido, a avaliação do padrão circadiano da PA em pacientes internados no hospital, não consiste em uma das indicações do método, mesmo com o objetivo de estudar desfechos específicos.

No presente estudo transversal e prospectivo, Turan et al. tiveram como objetivo avaliar, em pacientes hospitalizados com síndrome coronariana aguda (SCA), a relação entre a doença arterial coronariana (DAC) e menor descenso noturno da PA.^6^ Para avaliação da DAC, foi utilizado o escore SYNTAX, bem estabelecido para este fim. E para avaliação da PA foi adotada monitorização clínica, através de dispositivo automático em monitores a beira leito. Tais medidas foram realizadas a cada hora, nos períodos diurno e noturno. Os autores estabeleceram um protocolo próprio, considerando as médias dos valores horários de PA para 9 períodos noturnos (23:00 h às 07:00 h) e 15 períodos diurnos (8:00 h às 22:00 h), obtendo-se um único valor médio de PA diurna e noturna. Quanto à definição de descenso (dipping) presente, ausente ou reverso, os parâmetros foram os mesmos utilizados na MAPA.

Dentre os resultados obtidos, pode-se destacar que os pacientes internados com SCA, hipertensos e sem descenso noturno apresentaram escore SYNTAX mais alto. Além disto, o número de pacientes com escores altos foi significativamente maior no grupo de hipertensos sem descenso noturno, em comparação com o grupo com descenso. Apesar de não poder se estabelecer efeitos de causalidade, por se tratar de um estudo transversal, na análise por regressão logística mutivariada, o status de hipertensão sem descenso noturno (não-*dipper*) se apresentou como um preditor independente de alto escore SYNTAX.

Este é o primeiro estudo que avalia o comportamento da PA noturna em pacientes hospitalizados com SCA. Porém, outro estudo publicado por Mousa et al., já havia demonstrado uma associação entre hipertensão sem descenso, e DAC significativa em homens.^7^ Havia sido demonstrado que a ausência de descenso noturno correspondia a um marcador independente de risco para DAC, com dados obtidos pela MAPA, em pacientes clinicamente estáveis, e eletivos para realização de angiografia coronariana.

Algumas limitações, devidamente descritas por Turan et al., são bem importantes na análise do presente estudo. Dentre elas, destacam-se a falta da verificação da reprodutibilidade das medidas da PA, e a condição clínica dos pacientes avaliados, que pode ser muito distinta da condição habitual no dia a dia.^6^ Neste sentido, vale reforçar a menção pelos autores do estudo de Xu et al., que demonstrou boa correlação entre a medida manual tradicional da PA com esfigmomanômetro e a MAPA de 24 horas, na detecção de hipertensos sem descenso noturno, também em ambiente hospitalar.^8^

Sabemos que a MAPA é o padrão outro para a medida da PA, incluindo a avaliação do descenso noturno.^1,9^ Porém, estudos como este são importantes, para que tenhamos outras alternativas na avaliação circadiana da PA no ambiente hospitalar, e no estudo de sua correlação com desfechos. Novos estudos randomizados, e com avaliação da reprodutibilidade do método, poderão trazer maior respaldo para a prática clínica em ambiente hospitalar.
