# Supplementary material for: O Descenso Noturno Atenuado pode ser um Preditor de Gravidade e Complexidade da Doença Arterial Coronariana em Pacientes Internados com Síndrome Coronariana Aguda?
Source: Arq Bras Cardiol. 2022 Jul 7;119(1):85–6. [Article in Portuguese] doi: 10.36660/abc.20220401 (PMC9352131; doi:10.36660/abc.20220401)
Supplement: Translation

# Translation: Can Attenuated Nocturnal Dipping be a Predictor of the Severity and Complexity of Coronary Artery Disease in Hospitalized Patients with Acute Coronary Syndrome?

**Authors:** Andrea Pio-Abreu

**Affiliations:** 1 São Paulo SP Brazil Unidade de Hipertensão Arterial da Disciplina de Nefrologia, Universidade de São Paulo (USP), São Paulo, SP – Brazil

**Keywords:** Blood Pressure, Nocturnal Dipping, Arterial Hypertension, Acute Coronary Syndrome, Coronary Artery Disease

It is well established that there are significant prognostic implications resulting from the abnormal behavior of some 24-hour blood pressure (BP) parameters obtained through Ambulatory Blood Pressure Monitoring (ABPM).^1^ Among them, the abnormal behavior of the mean systolic blood pressure (SBP) and diastolic (DBP) in 24 hours, wakefulness and sleep, stand out.^2^ Specifically regarding the behavior of BP during sleep, it is already well established that the absence of nocturnal dipping has, independently, a significant impact on the increased cardiovascular risk.^3,4^ Other parameters that may have prognostic implications are early morning BP elevation and greater 24-hour BP variability.^1,5^

Some recommendations are essential for the success of the ABPM exam, among them, the orientation for the patient to maintain their usual activities during the monitoring day. In this sense, assessing the circadian pattern of BP in hospitalized patients is not one of the indications of the method, even intending to study specific outcomes.

In the present cross-sectional and prospective study, Turan T. et al. aimed to evaluate, in hospitalized patients with the acute coronary syndrome (ACS), the relationship between coronary artery disease (CAD) and lower nocturnal BP dips.^6^ To assess CAD, the SYNTAX score was used, which is well established for this purpose. And for BP assessment, clinical monitoring was adopted through an automatic device on monitors at the bedside. Such measurements were performed every hour, during the day and night. The authors established their own protocol, considering the averages of hourly BP values for 9 night periods (11:00 pm to 7:00 am) and 15 daytime shifts (8:00 am to 10:00 pm), obtaining a single mean daytime and nighttime BP value. Regarding the present, absent or reverse dipping definition, the parameters are the same used in the ABPM.

Among the results obtained, it can be highlighted that hospitalized patients with ACS, hypertensive and without nocturnal dipping had a higher SYNTAX score. In addition, the number of patients with high scores was significantly higher in the group of hypertensive patients without nocturnal dipping compared to the group with dipping. Despite not being able to establish causality effects because it is a cross-sectional study, in the multivariate logistic regression analysis, the status of hypertension without nocturnal dipping (non-dipper) was presented as an independent predictor of a high SYNTAX score.

This is the first study to assess the behavior of nocturnal BP in hospitalized patients with ACS. However, another study published by Mousa et al. had already demonstrated an association between hypertension without dipping and significant CAD in men.^7^ It was shown that the absence of nocturnal dipping corresponded to an independent risk marker for CAD, with data obtained by ABPM in clinically stable patients, and elective coronary angiography.

Some limitations, duly described by Turan T. et al., are very important in the present study's analysis. Among them, it stands out the lack of verification of the reproducibility of BP measurements and the clinical condition of the evaluated patients, which can be very different from the usual condition in everyday life.^6^ In this sense, it is worth reinforcing the mention by the authors of the study by Xu T. et al., who demonstrated a good correlation between the traditional manual BP measurement with a sphygmomanometer and the 24-hour ABPM in the detection of hypertensive patients without nocturnal dipping, also in a hospital.^8^

We know that ABPM is the gold standard for measuring BP, including the assessment of nocturnal dipping.^1,9^ However, studies like this are important so that we have other alternatives in the circadian assessment of BP in the hospital environment and the study of its correlation with outcomes. New randomized studies assessing the method's reproducibility may provide greater support for clinical practice in a hospital environment.
